# The Co-Constitution of Health Systems and Innovation: Comment on "What Health System Challenges Should Responsible Innovation in Health Address? Insights From an International Scoping Review"

**DOI:** 10.15171/ijhpm.2019.64

**Published:** 2019-07-31

**Authors:** Alexander Peine

**Affiliations:** Copernicus Institute of Sustainable Development, Utrecht University, Utrecht, The Netherlands.

**Keywords:** Health Innovation, Co-Constitution, Health Systems, Responsible Innovation

## Abstract

Lehoux et al provide a timely and relevant turn on the broad and ongoing discussion around the introduction of health technology and innovation. More specifically, the authors suggest a demand-driven approach to health innovation that starts from identifying challenges and demands at the health system level. In this commentary, I review a number of underlying implications of their study in relation to positions of technology push and techno-optimism, and to the narrow focus on health technology assessment on economic and clinical values. While Lehoux et al’s scoping review provides very relevant insights with the potential to drive further empirical research, it is less clear about its conceptual basis. In particular, the somewhat artificial distinction between health innovations and health systems is worth further scrutiny. I discuss some potential risks of this separation, and propose to more openly address the co-constitution of health, health systems and technology in future research along the lines suggested by Lehoux et al.

## Towards Demand-Driven Health Innovations

Lehoux et al^1^ provide a timely and relevant turn on the broad and ongoing discussion around the introduction of new health technologies. Most importantly, the paper contributes to the literature that has questioned a narrow perspective on clinical and economic values in health technology assessments, and it re-directs our attention to the broader health and innovation systems in which health technologies are embedded.^2^ Considering these systems is, according to Lehoux et al,^1^ pertinent in order to meaningfully implement responsible health innovations across a range of contexts, including countries with high, medium and low human development indices. More specifically, the authors suggest a demand-driven approach to health innovation that starts from identifying challenges and demands at the health system level. This, indeed, is an important complement to the many technology push perspectives on health innovation that thrive on often unnuanced framings of technological innovations as a panacea to societal challenges.^3,4^

It is particularly valuable that the paper challenges the dominant “more is better” approach to new treatments and health technologies. Here, often even small improvements of patient value are positioned as inherently “good,” although they may come with significant side effects, such as increases in health inequalities. The authors rightly point to the many risks that this perspective implies because its inherent techno-optimism might gloss over more delicate issues of affordability, acceptability and adaptability (p. 65). Indeed, if policy-makers were to follow suit, and continue to unquestioningly tie in health innovations with economic growth and a focus on healthcare costs, this implies significant risks that health innovations may contribute to the further commodification of healthcare and responsibilisation of patients.^3,5^ Next to the ethical questions this brings along, it will also hamper the equal distribution of health innovations among those that need it.

To rectify this, Lehoux et al highlight the need to design health innovations that purposefully support health systems. This is part and parcel of the emerging responsible innovation in health approach. However, according to Lehoux et al surprisingly little factual knowledge is available about the system level challenges that health innovations need to address in order to be responsible, and which health system challenges may even aggravate as result of health innovations. It is this very gap that the scoping review strives to close. If we are to direct health innovations into direction that “support health systems around the world” (p. 64), then we need to have at least a basic understanding of the range and scope of innovation challenges faced by health systems worldwide. And it is indeed perplexing that, so far, we do not have systematic insights in such challenges beyond more immediate economic and clinical values. A scoping review that maps the existing literature and takes stock of existing insights about health system challenges is highly valuable.

## Dynamizing Health System Challenges and Needs

The scoping review certainly is an important first step in grasping the implications of health innovation at the health system level. However, in order for such an agenda to thrive, I feel that a more explicit *conceptualization* of the entanglements of health innovations and health systems is also required. In this regard, I am not convinced that the framing of health innovations, on the one hand, and health systems, on the other, as two analytically separate and separable entities is helpful. At the theoretical level, this separation risks reinforcing the technology push logic – which essentially assumes technologies to have an “impact,” for better or worth, on otherwise independent social contexts^6^ – that the paper strives to eschew.

Against this background, I would like to highlight a number of conceptual issues that subsequent empirical research would need to consider in order to embrace the complexities of health innovation and its inherent and dynamic entanglement with the social and cultural values it is part of. In doing so, I draw on insights from Science and Technology Studies (STS), a domain that studies the mutual shaping of society and technology^[1]^.^7^

In STS a long-standing discussion concerns the “Collingridge” dilemma of innovation and technology assessment.^9-11^ It basically says that that innovation can be steered towards desirable directions relatively easily in early phases of development, when its consequences are not yet known. In later stages of innovations, when the “impact” of an innovation is known, influence is much more difficult. Against this background, the separation of context from innovation that Lehoux et al make seems to place too much emphasis on the needs of existing health systems as guidance for responsible innovation, where a more careful attention to *value dynamism* might be more appropriate.

Value dynamism is an issue that has recently been raised in the ethics of technology innovation, where scholars have begun to discuss that and how values and other ethical concerns like trust or privacy are dynamic and change alongside with innovations.^12,13^ For innovations, it is inherently problematic, then, to talk about an impact *on* specific aspects of an allegedly surrounding social system, or single out specific stable needs in relation to an innovation. For instance, Lehoux et al discuss the impact of health innovation on human resources. While this is indeed a crucial point in extending the existing focus on economic or clinical impact, it is also important to recognize how innovations may require a whole new range of tasks and job profiles that need to be mapped in order to fully understand impact on human resources. STS research on the introduction of new care technologies, for instance, has shown how the introduction of TeleCare systems bring into sight new job profiles, such as installers and call center employees.^14,15^ Lehoux et al do not fully exclude such fundamental shifts in health systems from their analysis. But in further research it needs to be clarified in how far the dynamic and mutually shaping relationships between innovations and health systems are taken on board conceptually.

## The Co-Constitution of Health Systems and Innovation

Recently, my colleagues and I working on ageing and technology have suggested a framework of co-constitution for the analysis and practical guidance of gerontechnological innovations.^4,6^ To that end, we have reviewed the emerging body of empirical literature in the field of what is increasingly referred to as *socio-gerontechnology* that explores ageing and technology as relational concepts across various domains, sites and scales.^6^ With the notion of co-constitution, we suggest that scholars avoid undue assumptions about ageing as a (more or less) fixed target for technological interventions, which is a tenet that underlies many conventional studies on ageing and technology.^16^ Rather, we suggest an approach that is attentive to the many ways in which the experience of ageing itself is constituted together with the increasing diffusion of technological innovations. So, our own studies in socio-gerontechnology have shown how technological innovation *creates* ageing and older people as much as it targets them.^17^ This is the case, for instance, when technology developers prioritize aspects of ageing that are easy to measure (such as the increased risk of falling), over those that are more difficult to grasp (such as social connectedness). Techological innovation then enacts older people as fallers, as much as it addresses the alleged risks of falling.^18^ The same is true for the life worlds of older people, where established values such as privacy or health are re-negotiated in interaction with newly introduced technologies, such as monitoring devices or a new medicine.^19,20^

In conclusion, therefore, I would like to suggest that further research into health innovation and health systems acknowledges the *co-constitution of health systems and innovation*. This will require doing away with the analytical separation between innovations and social context, to give way to an agenda for responsible innovation in health that is more attentive to the mutual shaping of innovations and health systems. As a most immediate consequence, it may be necessary to abond the widespread interventionist vocabulary that haunts current debates around ageing, health and technology, and that is expressed in terms like “impact,” “solution,” or “acceptance.” For sure, these terms have their merits in highlighting the relevance of thinking about the user side of health innovations. But they also assume, in one way or another, that there are stable and measurable effects of technologies on the lives of people and patients, or on the values and practices of health systems more broadly. A vocabulary that takes co-constitution to heart would rather put the evolving and generative entanglements of technologies with the values and practices of health systems center stage. As an empirical agenda, it allows us to study how such values and practices are constituted in relation to new technologies, rather than measuring an alleged impact on a pre-existing set of values and practices. This way, more nuanced approaches to responsible innovation in health will also become possible that are sensitive to the changing identities of both health systems and technologies as they evolve in relation to each other.

## Ethical issues

Not applicable.

## Competing interests

Author declares that he has no competing interests.

## Author’s contribution

AP is the single author of the paper.

## Footnotes

[1] To be sure, the lead author of the commented paper has pointed out the relevance of STS for understanding health innovation herself.^8^
